# Thyroxin displacement from single and combined thyroid hormone binding proteins by various test substances

**DOI:** 10.1007/s00204-026-04400-4

**Published:** 2026-06-27

**Authors:** J. Brandt, E. Fabian, A. Aktalay-Hippchen, L. Jedermann, R. Landsiedel

**Affiliations:** 1 Pharmacy, Pharmacology and Toxicology, Free University of Berlin, Berlin, Germany; 2 BASF SE, Experimental Toxicology and Ecology, Ludwigshafen am Rhein, Germany

**Keywords:** Serum protein binding, Thyroid hormone homeostasis, Thyroxine (T4) displacement, Endocrine disruption, Key event

## Abstract

The thyroid hormone system regulates physiological processes that are crucial for growth and metabolism. The homeostasis of thyroid hormones can be affected by test substances via multiple molecular interactions, one of them being the displacement of thyroid hormones from their binding proteins. The main thyroid hormone in the human bloodstream is L-thyroxine (T4), ≥ 99.5% of which is transported bound to three proteins: thyroxine binding globulin (TBG), transthyretin (TTR) and human serum albumin (HSA). To investigate the interactions of xenobiotics with the binding of T4 to these proteins, an in vitro thyroid hormone protein binding assay (THPB-assay) was developed. Samples containing 100 nM T4 were incubated with a T4-binding protein and increasing concentrations of a test compound, followed by removal of the unbound T4 via size exclusion chromatography and analysis of protein-bound T4. This assay allows for multiple adjustable parameters, including the choice of binding protein and the detection method for protein-bound T4, which can be extracted and measured via LC-MS or, when using ^125^I-T4, by a gamma counting. Eight test substances were tested for the displacement of T4 from TTR and TBG respectively. For interference with T4-TTR binding, Tetrabromobisphenol A was established as a positive control with an average measured IC_50_ of 287 nM. Displacement of T4 was observed for five more test substances, which displaced T4 from TTR with IC_50_ ranging from 101 nM for Tetrac and 121 nM for 2,4,6-tribromophenol (TBP) to 1056 nM for Genistein. 6-propylthiouracil and L-DOPA did not displace T4 from TTR. LC-MS analysis and gamma counting of ^125^I-T4 yielded similar results for each test substance. The five test substances which displaced T4 from TTR were additionally tested for displacement of T4 from TBG. Tetrac and Benziodarone displaced T4 with IC_50_ values of 3 µM and 22 µM, respectively, whereas the remaining compounds did not. Finally, a mix of TTR, TBG and HSA, in concentrations of 536 nM, 26.5 nM and 65.35 µM respectively, was tested with the potent T4 displacer Tetrac. Tetrac displaced T4 from this protein mix with an IC_50_ of 154 µM. Using the radioligand detection method, the T4 displacement of compounds from human and rat serum was tested to elucidate their effect in a physiological environment and identify potential species differences. Tetrac displaced T4 from human and rat serum at 10% serum concentration in samples, with IC_50_ values of 38 µM and 242 µM respectively. TBP displaced T4 from 10% rat serum with an IC_50_ of 1.3 mM. No displacement was observed in 10% human serum. At higher serum concentrations, no test substance was measured to displace T4 regardless of the species. While further research is essential, the insights regarding the absence of displacement at physiological serum concentrations challenge current risk assessment frameworks, which define changes in serum T4 levels as a key event in adverse outcome pathways related to the displacement of T4 from TTR.

**Supplementary Information:**

The online version contains supplementary material available at https://doi.org/10.1007/s00204-026-04400-4.

## Introduction

Thyroid hormones play a crucial role in fetal development, neuronal and skeletal development, energy homeostasis and adaptive thermogenesis among others (Bassett und Williams 2016; Dowling et al. 2000; Mullur et al. 2014). Thyroid hormone synthesis and expression is tightly regulated by the hypothalamic-pituitary-thyroid (HPT) axis. Dysregulations of the TH homeostasis, e.g., caused by exogenous substances, can lead to adverse effects on development, resulting in outcomes such as a reduced cognitive function in the offspring (Haddow et al. 1999). Multiple functions of the TH axis can be affected by exogenous substances, one of them being the transport of THs through the bloodstream by thyroid hormone binding proteins (THBPs).

The predominant serum TH, L-thyroxine or T4, is mainly distributed between three THBPs: thyroxine binding globulin (TBG), transthyretin (TTR) and human serum albumin (HSA). In humans, the bulk of T4 is bound to TBG, whereas only a minor fraction of T4 is carried by HSA. An intermediate amount of T4 is bound to TTR, however, TTR enables T4 transport across the choroid plexus towards the cerebrospinal fluid (CSF) and across the uterine-placental wall during pregnancy (Landers et al. 2009; Chanoine et al. 1992). These specialized roles of TTR have led to speculations about adverse outcomes in respect to neuronal and embryonal development in the case of endocrine disruption of T4-TTR binding. Displacement of T4 from TTR by exogenous compounds could result in a transient rise of free T4 levels, which in turn increases T4 tissue uptake and hepatic-biliary elimination, leading to decreased total T4 levels. Reduced T4 levels in offspring after PCB treatment have been observed in rodent studies, possibly caused by displacement of T4 from TTR (Darnerud et al. 1996). Whether these observations made in rodent studies can be extrapolated to humans is currently unclear, due to the different THBP composition between both species. The fact that the main T4 carrier in rodents is TTR, whereas TBG is not expressed in healthy adult specimens, may leave rodents more sensitive towards displacement of T4 from TTR (Rouaze-Romet et al. 1992b). Even though an accumulation of test substances interfering with the TH-axis has been observed in humans, a direct connection between a disruption of T4-TTR binding and thyroid related adverse outcomes could not be made as of yet (Soechitram et al. 2004). An interference of exogenous compounds with thyroid hormones binding to TTR and subsequent human neurodevelopmental effects is currently defined as an adverse outcome pathway (AOP) by the environmental, health and safety (EHS) programme of the organization for economic co-operation and development (OECD), namely as AOP 152 (Janus et al. 2024). However, it must be denoted that the current status of this AOP is still “under development”.

Several industrially produced chemicals have been identified as endocrine disrupting chemicals (EDCs) interfering with the TH axis resulting in adverse effects on the organism, including brominated flame retardants and polychlorinated biphenyls (PCBs) (Naveau et al. 2014; Jugan et al. 2010; Han et al. 2022). To elucidate the interference of test substances with thyroid hormone protein binding (THPB), numerous in vitro approaches have been developed. One of the first testing methods to examine T4-TTR binding uses radioactively labelled T4 and separation of TTR-bound T4 and free T4 by equilibrium dialysis (Raz and Goodman 1969). Since radioactivity measurements are relatively robust and sensitive, more recent testing methods still rely on radioactivity measurement (Hill et al. 2017). However, working with radioligands requires strict occupational and environmental safety measures, which is why methods not relying on radioactive T4 are preferred. Numerous TTR-binding assays with fluorescent probes have been developed such as T4 labelled with fluorescein isothiocyanate (T4-FITC) (Ren and Guo 2012). Alternatively, the fluorescent probe 8-anilino-1-naphthalenesulfonic acid ammonium salt (ANSA) selectively binds TTR (Montaño et al. 2012). Further testing methods include immobilized T4 on a biosensor chip, followed by a measurement of TTR or TBG binding to the immobilized T4 in the presence of test substances (Marchesini et al. 2008). Other approaches cover the interference of test substances with TTR-binding via quantification of TTR-bound T4 using TRβ-coupled luciferase reporter gene expression (Collet et al. 2020; Sprengel et al. 2021). Most testing methods focus on TTR as a THBP and ignore other THBPs in serum. Immunoassays focusing on the measurement of free T4 levels in serum are commonly used in a diagnostic setting, however, conditions affecting the binding properties of THBPs such as familial dysalbuminaemic hyperthyroxinaemia may lead to artefactual elevation of free T4 levels in these assays and consequent misdiagnoses (Khoo et al. 2020). Nevertheless, including other THBPs such as TBG, HSA or serum into the testing method can provide more physiologically relevant information about the dynamic interactions between test substances and T4-protein binding.

**Fig. 1:**
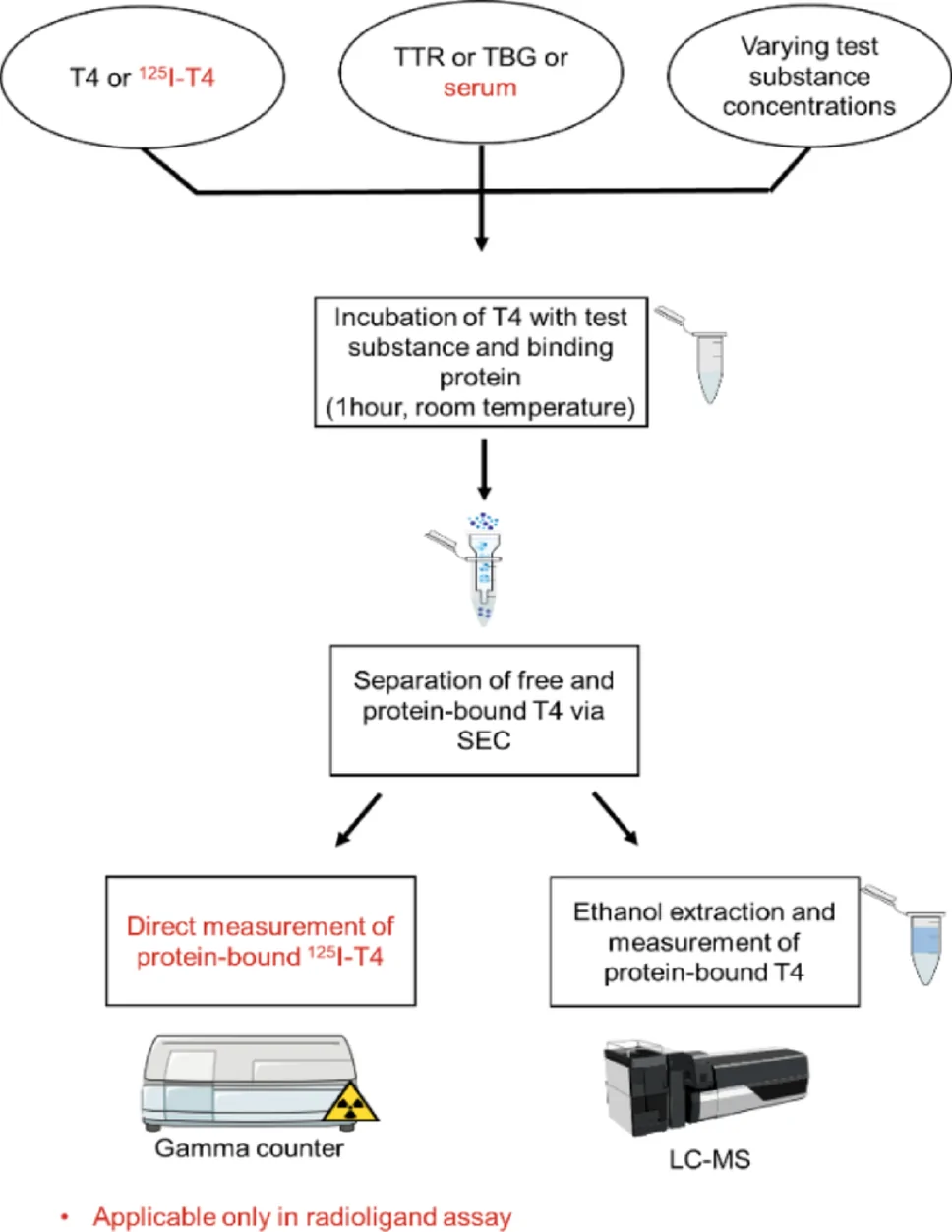
General workflow of the THPB-assay with multiple adjustable parameters. (T4—L-thyroxine, TTR—transthyretin, TBG—thyroxine binding globulin, SEC—size exclusion chromatography, LC-MS—liquid chromatography-mass spectrometry)

We established a thyroid hormone protein binding (THPB) assay to measure the displacement of T4 by test substances from different binding proteins: TTR, TBG, a mixture of THBPs, and total serum. After an incubation of the proteins with T4 and the test substance, the unbound T4 was separated from the protein bound T4 fraction using size exclusion chromatography (SEC). Protein bound T4 was quantified by LC-MS or as a radioligand using a gamma counter (Fig. 1). Eight test substances, 3,3´,5,5´-tetrabromobisphenol A (TBBPA), 2,4,6-tribromophenol (TBP), pentachlorophenol (PCP), 6-propylthiouracil (6-PTU), tetraiodothyroacetic acid (Tetrac), Benziodarone, Genistein and 3,4-dihydroxy-L-phenylalanin (L-DOPA), were tested for their interference with T4 binding to human TTR, TBG or mixtures of human THBPs as well as their ability to displace T4 from either human or rat serum proteins.

### Materials

#### Chemicals

All chemicals for the THPB-assay were purchased from Sigma-Aldrich unless stated otherwise. This includes transthyretin (TTR), thyroxine binding globulin (TBG), human serum, L-thyroxine (T4), the internal standard (IS) ^13^C_6_-L-Thyroxine, and all test substances. The rat serum was kindly provided by the Laboratory of Clinical Chemistry (BASF SE). TTR and TBG stock solutions and working solutions were prepared in tris buffer, made by mixing 1.51 g of Trizma base and 1.75 g of NaCl in 250mL HPLC-grade water (Macherey-Nagel) and adjusting the pH to 7.4 using 37% HCl in H_2_O (Analytichem GmbH). Stock solutions of test compounds as well as the stock solution for the reference compound for T4-TTR-assays, 3,3`,5,5`-tetrabromobisphenol A (TBBPA), were prepared in dimethyl sulfoxide (DMSO) (Table 1). Likewise, T4 stock solutions were prepared at 1.5mM in DMSO. All working solutions containing T4, test compounds or reference compounds were prepared in tris buffer containing 1% of DMSO (v/v). Calibration solutions were prepared with NaCl free tris buffer, made by mixing 0.30 g of Trizma base in 250mL of HPLC-grade water and adjusting the pH to 7.4. The extraction solution consisted of 15nM of IS and 0.16mM NaOH in ethanol.

#### Selection of test substances

The selection of test substances used to evaluate the THPB-assay was based on literature research and further aided using the in-house search engine Artor (BASF SE). Using Artor, compounds structurally similar to T4 were compiled and compounds of interest were selected as test substances. Most selected compounds were confirmed to displace T4 from serum binding proteins by previous research efforts (Mizuguchi et al. 2023; Marchesini et al. 2008; Radović et al. 2006). All test substances and reference substances used can be found in the table below, along with T4 for structural reference (Table 1).

**Table 1:** Name, Structure, CAS number and stock concentration of test substances. T4 and TBBPA included for structural reference

Substance	Structure	CAS number	Concentration stock solution [mM]
T4		51-48-9	1.5
3,3´,5,5´-tetrabromobisphenol A (TBBPA)		79-94-7	9.2
2,4,6-tribromophenol (TBP)		118-79-6	10
Pentachlorophenol (PCP)		87-86-5	10
6-propylthiouracil (6-PTU)		51-52-5	10
Tetraiodothyroacetic acid (Tetrac)		67-30-1	10
Benziodarone		68-90-6	10
Genistein		446-72-0	10
3,4-dihydroxy-L-phenylalanin (L-DOPA)		59-92-7	10

### Methods

#### THPB-assay with LC-MS analysis

All THPB-assay samples were pipetted in 1.5 mL LoBind tubes (Eppendorf^®^) to minimize adsorption of compounds to the surface of the sample tubes. To perform the THPB-assay, a series of test substance concentrations were incubated with 100 nM T4 and a fixed concentration of the respective THBP 260 nM for TTR-binding assay, 215 nM for TBG-binding assay) in 100 µL of tris buffer containing 0.2% DMSO (v/v). For test substance concentrations exceeding 10 µM, the DMSO concentration was changed to 1.1% (v/v) to enhance solubility. The concentration of the binding proteins was chosen based on the linear range of experimentally determined T4-protein binding curves (Fig. S2). Each test substance concentration was pipetted in triplicates. Under identical conditions, a reference compound (TBBPA for TTR-binding assay, Tetrac for TBG-binding assays) was incubated in a series of five concentrations. In addition, five negative controls, containing only T4 and the serum binding protein in tris buffer containing 0.2% DMSO, were incubated as well. Samples were incubated for 1 h at RT. To test the functionality of the SEC columns, one column test sample, containing only T4 and the test substance, was prepared. Finally, five calibration samples containing known concentrations of T4, ranging from 3 to 150 nM (final concentration: 1–50 nM) in NaCl-free tris buffer, containing 0.2% DMSO (v/v), were prepared to generate a standard curve for each assay run.

After the incubation, all samples containing serum binding proteins and the column test, were pipetted into Bio-Spin^®^ P-6 Gel Columns (Bio-Rad Laboratories, Inc), which were prepared previously according to manufacturer instructions. Samples were centrifuged at 1000 g for 4 min to separate the protein bound T4 from unbound T4. During the SEC, samples underwent a buffer exchange to 10 mM tris buffer. After centrifugation, 90 µL of all prepared samples were mixed with 180 µL of 15 nM IS in ethanol/NaOH to extract the protein bound T4. Each sample was then shaken for 1 min at 1000 rpm on a thermomixer at RT. The samples were then centrifuged at 13,000 g for 10 min to pellet the denatured protein. Finally, 90 µL of supernatant from each sample was transferred to a HPLC-vial and analyzed via LC-MS.

The LC-MS analysis was carried out with an Ultimate 3000 HPLC coupled with a Q Exactive Focus orbitrap mass spectrometer (Thermo Scientific™). Each sample was analyzed via single injection with an injection volume of 3 µL, using a YMC-Triart C18 ExRS 3.0 μm 50.0 × 3.0 mm chromatographic column (YMC). Solvent A consisted of 5% Acetonitrile (v/v) and 0.1% formic acid (v/v) in H_2_O. Solvent B consisted of 5% H_2_O (v/v) and 0.1% formic acid (v/v) in Acetonitrile.

The solvent composition during each run was defined as follows: 35% B at 0.0–4.5 min, 100%B at 4.6–8.0 min, 35%B at 8.1–12.0 min. T4 was analyzed using a H-ESI source in positive ion mode with a source temperature of 350 °C, a spray voltage of 2.8 kV, sheath gas flow of 35, auxiliary gas flow of 10 with a temperature of 280 °C and an S-lens RF-level of 70.

The MS data was analyzed using the Chromeleon Chromatography Data System (Thermo Scientific™). Output data was expressed as a ratio between the T4 signal and the IS signal. The output data of the calibration standards was used to generate a standard curve in Microsoft Excel (Microsoft^®^), enabling T4 quantitation. The output data of all samples and controls was normalized to the mean value of the negative controls. The normalized data of the samples were plotted in a semilogarithmic manner against the test substance concentrations in GraphPad Prism 8.0.2 (Graphpad Software Inc.). To calculate the IC_50_ of each test substance, the following non-linear regression equation with four parameters and a variable hill slope was used:

$$\:Y=\frac{Bottom+(Top-Bottom)}{1+\:\frac{IC50}{{X}^{HillSlope}}}$$

Two constraints were implemented into the equation, namely that the IC_50_ value is set to be above zero, whereas the bottom plateau was constrained to a value of zero.

#### Radioligand THPB-assay

The sample preparation of the radioligand THPB-assay was performed as described above, with some modifications. Trace amounts of ^125^I-T4 were added to cold T4 prior to the sample preparation. All samples were incubated and measured in 12 × 75 mm PS test tubes (Sarstedt). The activity of each sample was measured using a Multi Crystal LB 2111 Gamma Detector (Berthold Technologies) before and after the SEC. The activity ratio of each sample before and after the SEC was used as output data, which was expressed as the mass balance and calculated as follows:

$$\:Mass\:balance=100\%\:\times\:\:\frac{Activity\:postSEC}{Activity\:preSEC}$$

Using the mass balance values of each sample, concentration-response curves were established and the IC_50_ was calculated as described above. As opposed to the LC-MS based THPB-assay, where a standard curve was included in every assay, a standard curve was only established once to evaluate the linearity of the gamma count measurement (supplementary data Fig. S1).

#### T4-serum-assay

To gain further insight into the interaction of compounds from different classes in physiological conditions, we modified the THPB-assay to include serum as a test protein matrix. For the T4-serum-assays, it was decided that the radioligand assay was more suitable than the LC-MS based approach, since trace amounts of ^125^I-T4 could be spiked towards the innate T4 contained in the serum. With the LC-MS based method, significant amounts of T4 would have to be added to the serum to increase the sensitivity of the measurement. The innate T4 concentration of human and rat serum was determined via LC-MS with a modified extraction method to assess the change the T4 concentration upon spiking with ^125^I-T4 (Supplementary data Fig. S6, Table S3). Overall, for both human and rat serum two different concentrations were tested, namely 90% (v/v) and 10% (v/v) serum content in the incubate. For each concentration, the THPB-assay was carried out similar to the radioligand THPB-assay described above, however, ^125^I-T4 was spiked directly to the serum prior to incubation and no unlabeled T4 was added.

## Results

### T4-TTR-assay using LC-MS or gamma counting of radioactive ^125^I for T4 quantification

**Fig. 2:**
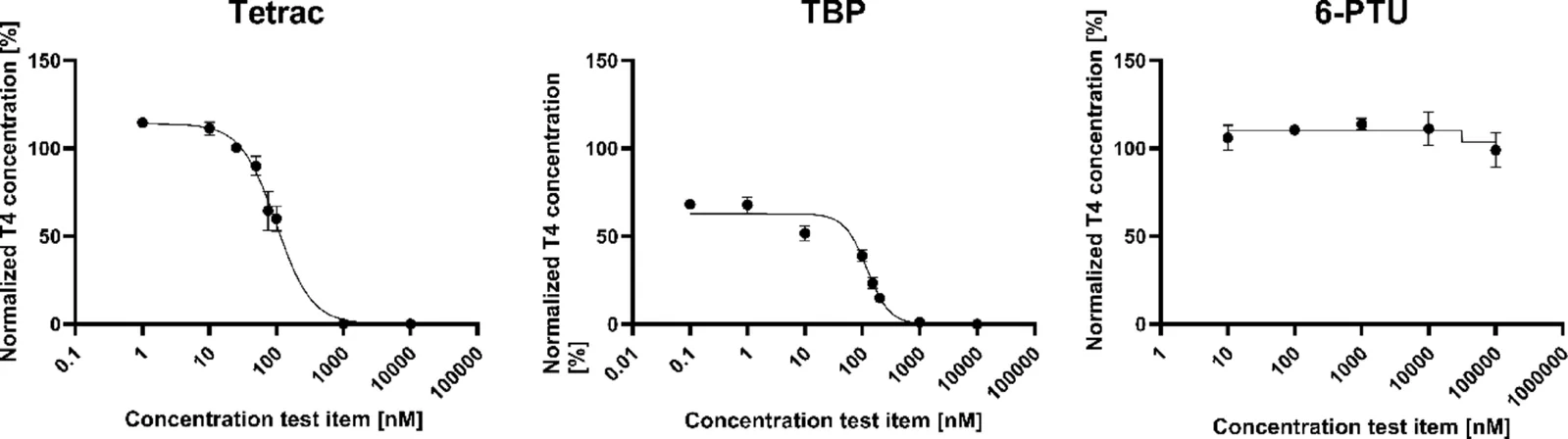
Concentration-response curves of tetrac, TBP and 6-PTU examined in the T4-TTR-assay with LC-MS analysis. Each test substance concentration was incubated and measured in triplicates. The T4 concentration, normalized to the negative controls of the respective assay run, and the corresponding standard deviations of the triplicates are depicted in a concentration-response curve in a semilogarithmic manner. Generated curve fits are depicted alongside the data. These graphs were generated using GraphPad Prism 8.1.2

To evaluate the potential of test substances to displace T4 from its binding proteins, the THPB-assay was first established with TTR as THBP. TBBPA was established as a positive control for T4-TTR assays with an average IC_50_ of 286.8 nM. The concentration-response curves for the test substances Tetrac, TBP and 6-PTU were depicted for graphical representation (Fig. 2). The complete set of concentration-response curves can be found in the supplementary information (Fig. S3–S5). The test substances TBP, PCP, Tetrac, Genistein and Benziodarone were identified as displacing T4 from TTR. No displacement of T4 could be observed for 6-PTU and L-DOPA. A small decline in T4 concentration observed with L-DOPA was not concentration dependent.

**Fig. 3:**
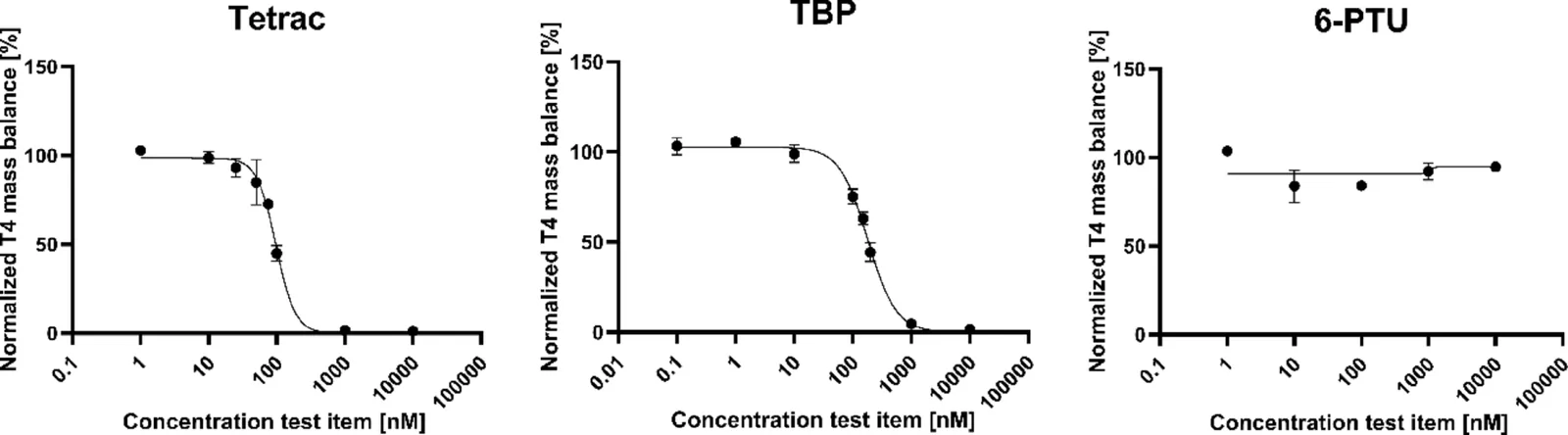
Concentration-response curves of Tetrac, TBP and 6-PTU examined in the T4-TTR-with gamma count analysis. Each test substance concentration was incubated and measured in triplicates. The T4 mass balance, normalized to the negative controls of the respective assay run, and the corresponding standard deviations of the triplicates are depicted in a concentration-response curve in a semilogarithmic manner. Generated curve fits are depicted alongside the data. These graphs were generated using GraphPad Prism 8.1.2

All test substances were tested using gamma counting of ^125^I-T4 in addition to the above described LC-MS quantification of T4 (Fig. 3). Both assays resulted in similar potencies to displace T4 from TTR for each test substance. Merely the IC_50_ of PCP was more than 2-fold higher when measured by gamma counting than by LC-MS measurement (Table 2).

**Table 2:** IC_50_ comparison of each test substance between the THPB-assay setups containing varying thyroid hormone binding proteins

Compound		Tetrac	TBP	PCP	Genistein	Benziodarone
IC_50_ [nM]	TTR (LC-MS)	101.2 (± 5.9)	120.6 (± 8.9)	179.1 (± 19.6)	1056 (± 252)	177.0 (± 24.5)
	TTR (radioligand)	97.5 (± 3.8)	180.2 (± 6.7)	437.7 (± 27.9)	1019 (± 120)	166.5 (± 10.2)
	TBG	3,234 (± 0.300)	N.A.	N.A.	N.A.	21,543 (± 3400)
	Protein mix	4.29*10^5^ (± 1.24*10^5^)	No data	No data	No data	No data
	Human serum 10%	3.85*10^4^ (± 9,900)	No data	No data	No data	No data
	Rat serum 10%	2.42*10^5^ (± 1.24*10^5^)	1.31*10^6^ (± 5.21*10^5^)	No data	No data	No data

Standard error of mean (SEM) is provided alongside the calculated IC_50_. IC_50_ containing the value “N.A.” indicate that a displacement was not observed and an IC_50_ could not be calculated

### T4-TBG-assay

**Fig. 4:**
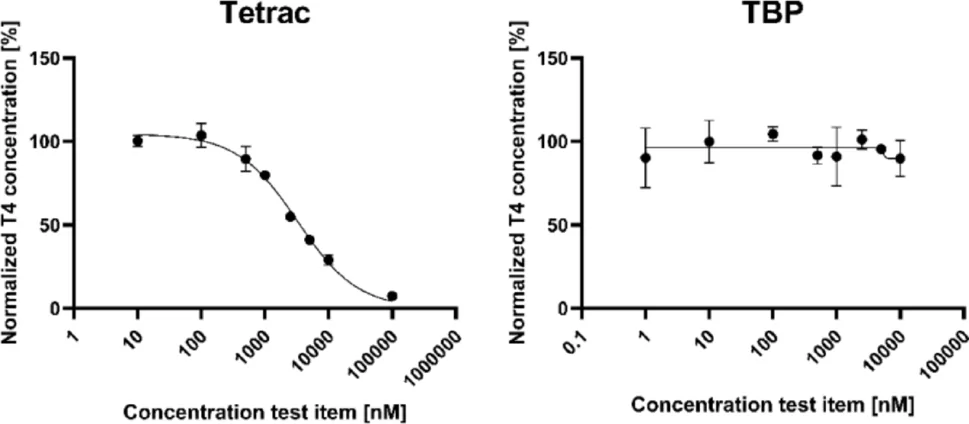
Concentration-response curves of Tetrac and TBP examined in the T4-TBG-assay with LC-MS analysis. Each test substance concentration was incubated and measured in triplicates. The T4 concentration, normalized to the negative controls of the respective assay run, and the corresponding standard deviations of the triplicates are depicted in a concentration-response curve in a semilogarithmic manner. Generated curve fits are depicted alongside the data. These graphs were generated using GraphPad Prism 8.1.2

Of all tested substances, only Tetrac and Benziodarone displaced T4 from TBG with IC_50_ of 3.2 µM (± 0.0.3 µM) and 21.5 µM (± 3.4 µM), respectively. These IC_50_ were, respectively, 31 and 122 times higher than those observed in the T4-TTR-assay. TBP, PCP and Genistein did not displace T4 from TBG (Fig. 4).

### T4-protein-mix-assay

**Fig. 5:**
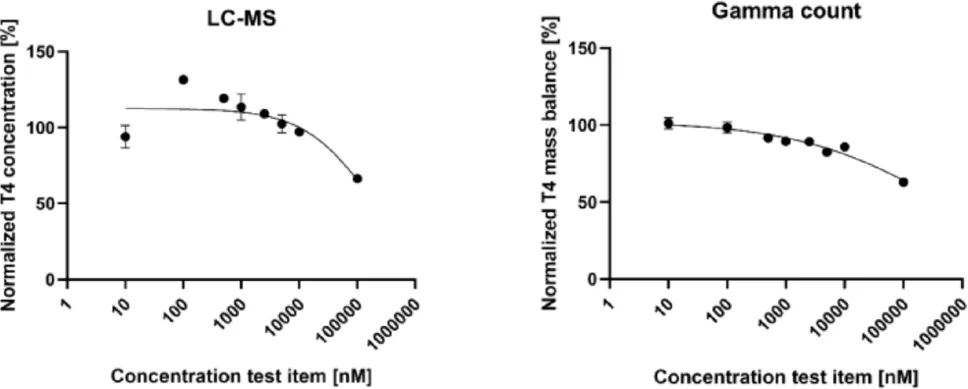
Results of the T4-protein-mix-assay with Tetrac as a test substance. Tetrac was tested with LC-MS analysis (left) and gamma count analysis (right). Each concentration was incubated and measured in triplicates. The T4 concentration (LC-MS) or mass balance (gamma count), normalized to the negative controls of the respective assay run, and the corresponding standard deviations of the triplicates are depicted in a concentration-response curve in a semilogarithmic manner. Generated curve fits are depicted alongside the data. These graphs were generated using GraphPad Prism 8.1.2

**Fig. 6:**
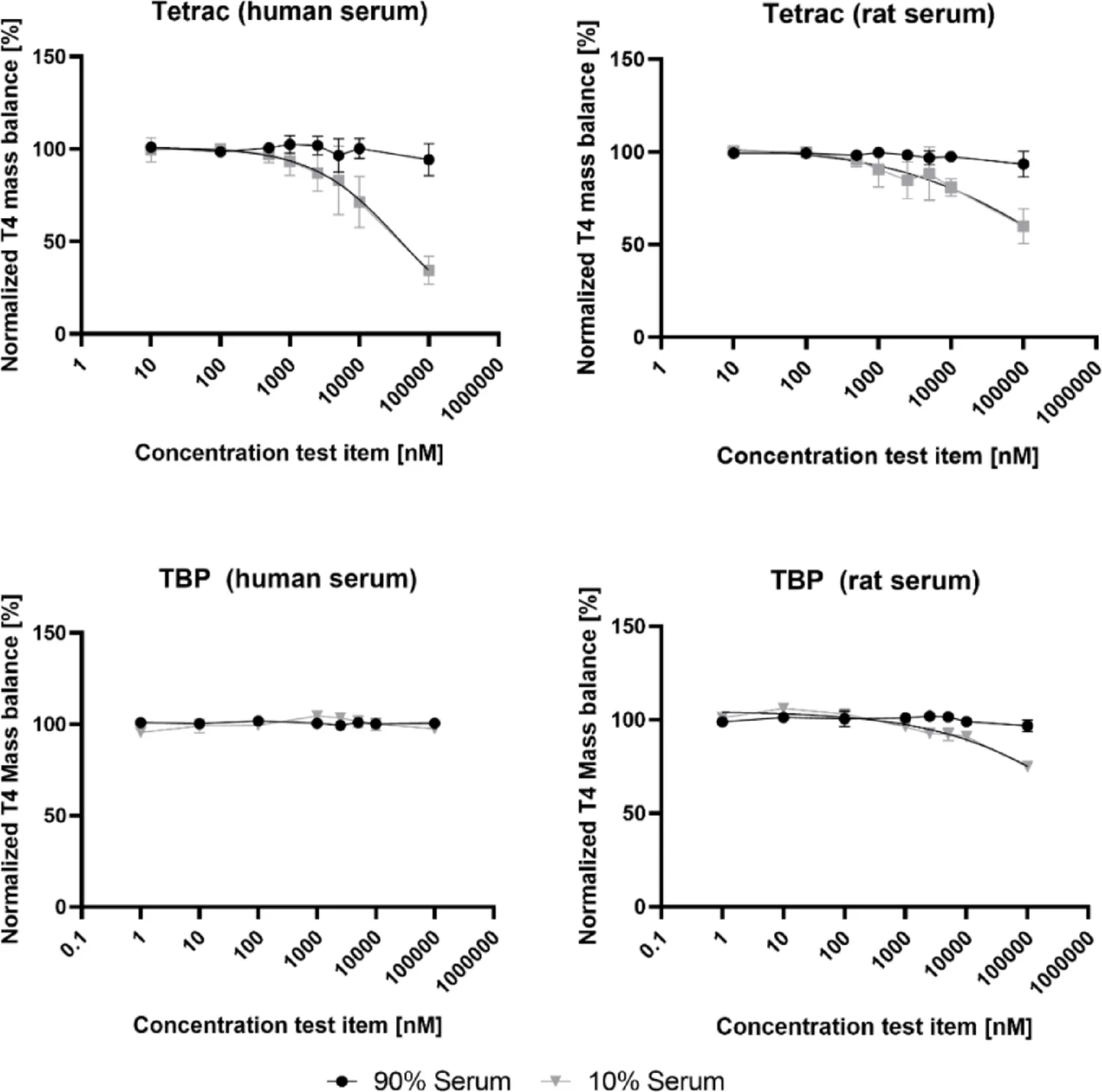
Results of the T4-serum-assay with gamma count analysis. Tetrac and TBP were tested with human and rat serum at 90% (black) and 10% (grey) serum concentrations respectively. Each test substance concentration was incubated and measured in triplicates. The T4 mass balance, normalized to the negative controls of the respective assay run, and the corresponding standard deviations of the triplicates are depicted in a concentration-response curve in a semilogarithmic manner. Generated curve fits are depicted alongside the data. These graphs were generated using GraphPad Prism 8.1.2.

To assess all relevant T4 binding proteins the THPB-assay was performed with a mix of TBG, TTR and HSA with concentrations in the incubate of 1.43 mg/L, 29.48 mg/L and 4378 mg/L, respectively. This represents approximately 10% of the physiological concentrations in human serum (Arques 2018; Burr et al. 1977; Ingenbleek and Bernstein 2015). Tetrac was selected for this assay as the most potent T4 displacer among the test substances. Its IC_50_ were 154 µM (± 56 µM) and 429 µM (± 124 µM) using quantification by LC-MS and gamma-counting of ^125^I-T4, respectively (Fig. 5). The IC_50_ were extrapolated as a maximum test substance concentration of 100 µM (100000 nM) was tested and did not achieve 50% T4 displacement (Fig. 6). The IC_50_ to displace T4 from THBPs was higher when using the mix of TTR, TBG and HSA compared to TTR or TBG alone.

### T4-serum-assays

The THPB-assay using gamma-counting of ^125^I for quantification was performed with serum providing the THBPs. Both human serum and rat serum were used each with two serum concentrations in the assay, 10% (v/v) and 90% (v/v). Literature-derived concentration ranges of TTR, TBG and albumin for the respective serum concentrations can be found in Table 4. TBP, observed to displace T4 from TTR, and Tetrac, observed to displace T4 from both TTR and TBG, were selected as test substances. Tetrac had a low potency to displace T4 from the serum of both species at a protein concentration of 10%, with human serum being slightly more affected than rat serum. TBP had a small effect on T4 bound to rat serum at a concentration of 10% in the assay but no effect of T4 bound to human serum. When using 90% serum concentration, no displacement of T4 from human or rat serum by either test substance could be observed.

**Table 3:** Concentrations of thyroid hormone binding proteins in the binding assays

		Protein concentration in the assay [mg/L]		Data source
		10% serum	90% serum	
Human serum	TTR	25–33	225–297	(Ingenbleek and Bernstein 2015)
	TBG	1.07–1.32	9.63–11.88	(Burr et al. 1977)
	Albumin	3500–5000	2900–3100	(Arques 2018)
Rat serum	TTR	35–41.2	315–370.8	(Bleiberg et al. 1985)
	TBG	Not detectable (adult rats)		(Young et al. 1985; Savu et al. 1991)
	Albumin	31,500–45,000	26,100–27,900	(Zaias et al. 2009)

Concentrations were calculated from data reported in the literature

## Discussion

### T4-TTR-assay

With the exception of L-DOPA all of the test substances tested in the T4-TTR-assay were already tested in previous studies(Marchesini et al. 2008; Mizuguchi et al. 2023; Jedermann 2023). L-DOPA is a precursor of tyrosine and T4; consequently, these molecules share some structural similarities. Furthermore, L-DOPA was observed to interact with the HPT-axis through inhibition of TSH secretion upon systemic administration in rats (Krulich et al. 1977). However, other interactions of L-DOPA with the HPT-axis have not been researched to the best of our knowledge and, in this study, L-DOPA did not displace T4 from TTR. 6-PTU, is interfering with the TH axis as a potent TPO inhibitor, but did not displace T4 from TTR in accordance with previous studies (Jedermann 2023). All other test substances displaced T4 from TTR, with the T4 analogue Tetrac being most potent, and the isoflavone Genistein least potent. Marchesini and colleagues tested Tetrac, TBP, PCP and Genistein in their T4-TTR binding study using immobilized T4 on a biosensor chip. Consistent with our results, Tetrac had the highest potency among the four test substances in displacing T4 from TTR, followed by TBP and PCP, while Genistein exhibited the lowest potency. Benziodarone was tested by Mizuguchi and colleagues for T4 displacement from TTR using the fluorescent probe ANS. It was identified as a displacer of but no IC_50_ was determined. In our study, Benziodarone displaced T4 from TTR with a relatively high potency comparable to TBP and PCP.

The displacement of T4 from TTR by all seven test substances was quantified by LC-MS and by gamma counting of ^125^I-T4. The concentration-response curves obtained with both quantification methods were generally similar; the gamma counting provided overall less variability of the results. Both quantification methods are suitable. The LC-MS method is, however, less demanding in occupational and environmental safety measures and could be the method of choice. The use of ^125^I-T4 may be justified in cases where the LC-MS assay does not deliver reliable results due to interfering test substances or sample matrix. As both quantification methods may be used by different labs, it is worth noting that the results obtained with both methods are comparable.

### T4-TBG-assay

Of the five test substances, only Tetrac and Benziodarone displaced T4 from TBG. Tetrac displaced T4 from TBG with the highest potency of all test substances making it a suitable positive control for future T4-TBG-assays. While numerous substances were reported to interfere with T4 binding to TTR, T4 displacement from TBG was only observed for a few substances (Marchesini et al. 2008; Lans et al. 1994). This is likely due to the high binding affinity of T4 towards TBG (~ 1*10^10^M^−1^) as compared to TTR (~ 1*10^8^M^−1^) (Pappa et al. 2015). While Tetrac has been reported to interfere with T4-TBG binding, Benziodarone, to the best of our knowledge, had not yet been tested in a T4-TBG binding assay.

### T4-protein-mix-assay

We have tested Tetrac in a mix of three THBPs, TTR, TBG and HSA, with their concentrations in the incubate representing approximately 10% of human physiological serum concentrations of the respective THBPs. The THPB-assay using LC-MS for quantification resulted in recoveries above 100%, which indicates artefacts due to interfering substances in the sample matrix (supplementary data Table S4). For this reason, quantification by gamma counting of ^125^I-T4 was used because radioactivity measurements are generally less susceptible to interfering substances compared to LC-MS. The IC_50_ of 429 µM ± 124 µM obtained by quantification with gamma counting of ^125^I-T4 is therefore more reliable than the lower IC_50_ obtained by LC-MS quantification. Yet even this IC_50_ is extrapolated as 50% displacement was not observed by the maximum Tetrac concentration used in both assays. The results indicate a considerably lower potency of Tetrac to displace T4 from the mix of THBPs as compared to single TTR or TBG, despite the protein concentrations being in a comparable range. However, the HSA concentrations are orders of magnitude greater than the TTR and TBG concentration in this THBP-mix which is reflecting the relative concentrations in serum. HSA can compete with TTR and TBG for binding either the test substance or the T4 displaced from TTR or TBG by the test substance. Even with a lower binding affinity of HSA compared to TTR and TBG, HAS may bind relatively large amounts of the test substance or T4 due to its higher concentration in serum, in accordance with the law of mass action. Consequently, this markedly lower observed potency of Tetrac can be attributed to HSA preventing a rise in free T4 levels in the presence of compounds disrupting T4-TTR- and T4-TBG-binding.

### T4-serum-assay

T4-displacement from a single THBP protein, TTR or TBG, is providing information on the specific interactions of the test substances with these proteins. However, the actual interference of test substances with T4 protein binding in the body and hence the effect on the protein-bound and unbound fraction of T4 in serum, is more closely reflected by a THBP-mix or actual serum being used in the assays. TBP and Tetrac were both tested with human and with rat serum. In the THBP-specific assays, Tetrac and TBP displaced T4 from TTR but only Tetrac also displaced T4 from TBG. Human and rat serum differ in their TTR- and TBG concentrations: Whereas TTR concentrations are comparable between both species with around 250–330 mg/l and 350–412 mg/l in humans and rats respectively, TBG levels are nearly undetectable in young adult rats. Only during a short postnatal period, dietary restrictions, and during senescence, rat TBG levels are markedly increased. In humans, TBG levels can range from 10.7 to 13.2 mg/l (Bleiberg et al. 1985; Burr et al. 1977; Ingenbleek and Bernstein 2015; Rouaze-Romet et al. 1992a).

Assay incubations containing 10% serum (v/v) correspond to the THBP-mix of TTR, TBG and HSA as tested in the respective assay (described above). At this serum concentration, Tetrac displaced some T4 from human serum proteins with a markedly lower IC_50_ than determined in the T4-Protein-Mix-assay. However, accounting for the different concentrations of T4 in the incubate used in both assays (100 nM in the THBP-mix, ~ 15 nM in human serum assay), the potency of Tetrac is comparable between both assays. TBP did not displace T4 at this 10% human serum concentration in the assay. In contrast to human serum, both test substances could displace T4 in rat serum at a serum concentration of 10% in the assay. The potency of Tetrac was greater than the potency of TBP to displace T4 in rat serum, similar to the findings of the T4-TTR-assays. Tetrac had a higher potency to displace T4 in human serum than in rat serum, even though the potency of Tetrac to displace T4 from TTR is greater than its potency to displace T4 from TBG. In human serum the largest fraction of T4 is bound to TBG. Approximately 20–50% of TBG is occupied by T4 in human serum, based on previously mentioned human TBG concentration ranges and a T4 concentration of 50–120 µg/L (Stockigt 2010; Burr et al. 1977). Conversely, the majority of T4 binding sites of TTR in human serum remain unoccupied, with approximately 0.5% of TTR bound to T4, based on our measured T4 concentration, and TTR concentrations and TTR-bound fraction of T4 derived from literature (Pappa et al. 2015; Ingenbleek and Bernstein 2015). Similarly, in rat serum, the occupancy of TTR by T4 can range between 0.7 and 1.1% (Davis et al. 1970; Bleiberg et al. 1985). Hence the displacement of T4 by test substances in the TTR-assay may have much less impact on the total T4 protein binding in serum.

Notably, with 90% serum protein in the assay, neither Tetrac nor TBP displaced T4. Most probably, high concentrations of HSA and possibly other serum proteins prevent a rise in free T4 levels. This should be considered when assessing key events of AOP related to thyroid hormone homeostasis. The AOP no. 152 “Interference with thyroid serum binding protein transthyretin and subsequent adverse human neurodevelopmental toxicity” considers displacement of T4 from TTR as a key event which would increase the fraction of unbound serum T4. Looking at the T4 displacement from TTR in isolation may, however, be misleading. Displacement of T4 from TTR by test substances is attenuated in the presence of other serum proteins, because only a small fraction of T4 is bound to TTR compared to other proteins in serum and most of the test substance may bind to serum proteins without displacing T4.

Even if T4 was displaced from TTR, it could be redistributed to HSA, resulting in unchanged free serum T4 concentrations. Whether HSA can compensate for the high affinity T4 binding of TTR and its specialized roles in embryonal and neuronal development requires further research and may be substance specific. However, previous research efforts with test substances included in the present study suggest specific binding of test substances to TTR and a consequent redistribution to HSA is the more predominant mechanism (Mizuguchi et al. 2023; Radović et al. 2006). In any case, other serum proteins besides TTR, their concentration and T4 binding capacity and affinity need to be considered when assessing the fraction of free T4 in serum, its impact on thyroid hormone homeostasis and its potential to lead to adverse effects on the organism.

## Conclusion

We established a thyroid hormone protein binding (THPB) assay using LC-MS or gamma counting of ^125^I to quantify T4. Both quantification methods provided similar results. The test substance or serum components can however interfere with the LC-MS method; the radiometric method was more robust.

The IC_50_ data derived from the concentration-response curves can be used to characterize the potency of a test substance to displace T4 from the THBP used in the assay. The results obtained with test substance which have been tested previously in other assays are consistent with the respective results. Tetrac was most potent to displace T4 from TTR among the eight test substances, with an IC_50_ of 101.2 (± 5.9) and 97.5 (± 3.8) nM measured by LC-MS and radiometry, respectively. It was also most potent among the test substances to displace T4 from TBG with an IC_50_ of 3.2 (± 0.0.3) µM.

THBP-assays with single proteins with high affinity for T4, like TTR and TBG, are useful to gain insight into the interference of test substances with these proteins. In serum, however, other proteins co-determine the fractions of unbound and protein-bound T4 in the presence of test substances. Test substances were less potent to displace T4 from proteins in a mix of TTR, TBG and HSA representing 10% of the serum protein concentration, compared to assays only using either TTR or TBG. Similar results were obtained when using 10% serum (v/v) in the assay. This indicates that these three proteins together are key for T4 binding in serum. When using 10% of serum in the assays, differences of Tetrac and TBP to displace T4 from proteins in human and in rat serum were observed. When using 90% serum concentration in the assays, however, no displacement of T4 by the test substances was measured. Most likely the high HSA concentration compared to TTR and TBG in serum is outweighing any effect of test substances on these specific T4-binding proteins. Hence, the assessment of T4-displacement by test substances from TTR or TBG alone may be misleading when assessing consequences for thyroid homeostasis and adverse outcome pathways should not be restricted to only one TBHP for the key event of increasing free T4 in serum. Rather, assays assessing T4-displacement from THBP should use relevant protein mixtures and concentrations.

## Supplementary Information

Below is the link to the electronic supplementary material.

Supplementary file1 (DOCX 5817 kb)

## Abbreviations

6-PTU: 6-propylthiouracil
ANSA: 8-anilino-1-naphthalenesulfonic acid ammonium salt
AOP: Adverse outcome pathway
CALUX: Chemical activated luciferase gene expression
CSF: Cerebrospinal fluid
DMSO: Dimethyl sulfoxide
EDC: Endocrine disrupting chemical
EHS: Environmental, health & safety
EtOH: Ethanol
FITC: Fluorescein isothiocyanate
HSA: Human serum albumin
HESI: Heated electrospray ionization
HPLC: High performance liquid chromatography
HPT: Hypothalamic–pituitary–thyroid
IC_50_: Half maximal inhibitory concentration
LC: MS–Liquid chromatography—mass spectrometry
L-DOPA: 3,4-dihydroxy-L-phenylalanin
LOD: Limit of detection
LOQ: Limit of quantification
MIE: Molecular initiating event
n.a.: not available
OECD: Organization for economic co–operation and development
PBDE: Polybrominated diphenyl ethers
PBPK: Physiologically based pharmacokinetics
PCB: Polychlorinated biphenyls
PCP: Pentachlorophenol
PS: Polystyrene
RF: Radio frequency
RSD: Relative standard deviation
SEC: Size exclusion chromatography
T3: Triiodothyronine
T4: Thyroxin or tetraiodothyronine
TBBPA: Tetrabromobisphenol A
TBG: Thyroxine binding globulin
TBP: Tribromophenol
THBP: Thyroid hormone binding protein
THPB: Thyroid hormone protein binding
TPO: Thyroid peroxidase
TRβ: Thyroid hormone receptorβ
TSH: Thyroid stimulating hormone or thyrotropin
TTR: Transthyretin
WS: Working solution
