# Supplementary material for: Thyroxin displacement from single and combined thyroid hormone binding proteins by various test substances
Source: Arch Toxicol. 2026 Jun 27;100(11):5123–33. doi: 10.1007/s00204-026-04400-4 (PMC13694536; doi:10.1007/s00204-026-04400-4)
Supplement: Supplementary file 1 [file 204_2026_4400_MOESM1_ESM.docx]

# Supplementary Data

**Biospin Columns instructions**

Instructions for Use:

1 Invert the column sharply several times to resuspend the settled gel and remove any bubbles.

2 Snap off the tip and place column in a 2.0 ml microcentrifuge tube (included). Remove cap. Allow the excess packing buffer to drain by gravity to top of gel bed. (If column does not begin to flow, push cap back into column and remove). Discard the drained buffer, then place the column back into 2 ml tube. However, if placing column into 12 x 75 mm test tube, centrifuge immediately.

3 Centrifuge for 2 min in a swinging bucket centrifuge at 1,000 x g (see Centrifugation Notes section) to remove the packing buffer. Discard the buffer.

4 Place the column in a clean 2.0 ml microcentrifuge tube or 12 x 75 mm test tube. Carefully apply the sample (20–100 µl) directly to the center of the column. Application of more or less than the recommended sample volume may decrease column performance. 5 After loading sample, centrifuge the column for 4 min at 1,000 x g.

6 Following centrifugation, the purified sample is now in Tris or SSC buffer. Molecules smaller than the column’s exclusion limit will be retained.

7 Properly dispose of the used column.

**Validation of the LC-MS measurement method:**

The LC-MS measurement method was validated in terms of linearity, accuracy and precision, the limit of detection (LOD) and the limit of quantification (LOQ). The acceptance criteria were derived from established in-house validation guidelines.

Linearity: The samples for the standard curve used to validate linearity were prepared identical to the calibration samples used in the experiments, with 5 samples in a final concentration range of 1-50nM of T4. The ratio of the T4 signal to the IS signal was plotted against the T4 concentration of each sample. The linearity was expressed as the correlation coefficient R^2^. A coefficient of determination (R^2^) > 0.99 and back calculated accuracies of the calibration solutions in the range from 85 % - 115 % (80% -120% for the lowest concentration) were required to meet the acceptance criteria.

The standard curve used to validate the linearity of the LC-MS measurement parameters resulted in a coefficient of determination (R^2^) of 0.999 with back-calculated accuracies of 86.4-101.4% (Table S1). Both linearity parameters sufficiently met the acceptance criteria.

Table S1: Linearity parameters derived from the validation experiment. Parameters were compared to their respective acceptance criteria.

| Parameter | | Results from standard curve | Acceptance criteria |
| --- | --- | --- | --- |
| Linearity | R^2^ | 0.999 | >0.99 |
|  | Slope | 0.030 | n.a. |
| Back-calculated accuracy [%] | 1nM | 86.42 | 80-120 |
|  | 5nM | 91.17 | 85-115 |
|  | 10nM | 97.83 |  |
|  | 25nM | 101.38 |  |
|  | 50nM | 99.85 |  |

Accuracy and precision: The accuracy and precision of the assay setup were measured at three different concentrations of T4 (33.3nM, 13.33nM and 5nM). 6 replicates were prepared for each concentration level. TTR was added to all samples which were prepared under the assay conditions described above. The T4 concentration during incubation was 100nM, 40nM and 15nM respectively. The accuracy of each analysis was calculated with reference to the calibration curve used to validate linearity. Accuracy was expressed as a percentage of the measured T4 concentration relative to the nominal value. A mean accuracy of each measured fortification level in the range of 70-110% was required to meet the acceptance criteria. Lower recoveries may be accepted in technically difficult situations, provided the precision data is acceptable. The relative standard deviation (RSD) reported at each fortification level represents the precision of the method used. The RSD for each fortification level must be ≤ 15% for the method to be considered precise.

The calculated accuracy and precision for each fortification level can be found in the table below (Table S2). The precision of each fortification level ranged from 9.69-11.01%. The recovery ranged from 69.97-86.84%, in which the fortification level of 13.33nM resulted in a recovery rate outside of the acceptance criteria. However, given the fact that the precision was in an acceptable range, this recovery rate may still be accepted.

Table S2: Accuracy and precision parameters derived from the validation experiment. Parameters were compared to their respective acceptance criteria.

| Parameter | | Measured value | Acceptance criteria |
| --- | --- | --- | --- |
| Recovery [%] | 33.33nM | 72.07 | 70-110 |
|  | 13.33nM | 69.97 |  |
|  | 5nM | 86.84 |  |
| RSD [%] | 33.33nM | 9.69 | <15 |
|  | 13.33nM | 10.48 |  |
|  | 5nM | 11.01 |  |

LOD and LOQ: The LOD and LOQ were derived from the signal-to-noise (s/n) ratio. The integrated signal of a blank sample was used to determine the noise signal. The s/n-ratio of the LOD and LOQ was expressed as T4 concentrations in respect to the slope of the calibration curve:

*LOD= 3*Area(Noise)/Area(IS)/b*

*LOQ= 10*Area(Noise)/Area(IS)/b*

Since an internal standard signal is not present in blank samples, the LOD and LOQ were calculated with the IS peak area of each calibration sample respectively. The highest calculated LOD and LOQ were chosen as the final validation parameters. This resulted in an LOD and LOQ of 0.12 nM and 0.42 nM respectively.

**Standard Curve Gamma count measurement**

Figure S1: Calibration curve of ^125^I-T4. Measured gamma activity plotted against the nominal activity of calibration solutions. The linear regression equation and the corresponding determination coefficient (R^2^) are pictured below.

Figure S2: Binding curve of T4 and the serum binding proteins TBG, TTR and HSA (preliminary data). The linear ranges of the T4 binding curves were used to determine a suitable protein concentration for the respective THPB-assays.

Figure S3: Results of the T4-TTR-assay with LC-MS analysis. Each test substance concentration was incubated and measured in triplicates. The T4 concentration, normalized to the negative controls of the respective assay run, and the corresponding standard deviations of the triplicates are depicted in a concentration-response curve in a semilogarithmic manner. Generated curve fits are depicted alongside the data. These graphs were generated using GraphPad Prism 8.1.2.

Figure S4: Results of the T4-TTR-assay with gamma count analysis. Each test substance concentration was incubated and measured in triplicates. The T4 concentration, normalized to the negative controls of the respective assay run, and the corresponding standard deviations of the triplicates are depicted in a concentration-response curve in a semilogarithmic manner. Generated curve fits are depicted alongside the data. These graphs were generated using GraphPad Prism 8.1.2.

Figure S5: Results of the T4-TBG-assay with LC-MS analysis. Each test substance concentration was incubated and measured in triplicates. The T4 concentration, normalized to the negative controls of the respective assay run, and the corresponding standard deviations of the triplicates are depicted in a concentration-response curve in a semilogarithmic manner. Generated curve fits are depicted alongside the data. These graphs were generated using GraphPad Prism 8.1.2.

**Serum T4 quantification**

The T4 concentration of each serum species was determined by spiking 90µL of serum per sample with 10µL of T4 containing solution with final T4 concentrations ranging from 10-150nM per sample, along with a solvent-spiked sample. To precipitate the serum and extract the T4, 900µL of acetonitrile spiked with 11.1nM of IS was added, resulting in a final IS concentration of 10nM. The final concentration of the spiked T4 in the supernatant ranged from 1-15nM. After the addition of acetonitrile, the samples were shaken on a thermomixer for 1min at 1,000rpm, followed by a centrifugation step at 13,000g for 10min. The T4 concentration in the supernatant was then measured by LC-MS. The measured T4/IS signal ratio of each sample was used to generate a standard curve, from which the T4 concentration of each serum species could be extrapolated through the intercept on the abscissa. It is important to note that, due to technical reasons, the human serum samples spiked with 5nM and 15nM of T4 did not yield a measurable T4 signal and were thus omitted from this experiment.

Figure S6: Serum quantification of T4 in human serum and rat serum. T4 concentrations of T4 spiked serum samples ranging from 0-15 nM (0-10 nM in human serum) were measured via LC-MS. Based on the linear regression equation, the innate T4 concentrations of both serum species were determined by calculating the intercept on the abscissa.

Table S3: Parameters of the linear regression line based on measured T4 concentrations in T4 spiked human and rat serum. Based on these parameters, the T4 sample and serum concentration were calculated.

|  | **slope** | **y-intercept** | **T4 concentration in sample [nM]** | **T4 serum concentration [nM]** |
| --- | --- | --- | --- | --- |
| **Human serum** | 0.0220 | 0.2951 | 13.41 | 149.04 |
| **Rat serum** | 0.0226 | 0.1995 | 8.83 | 98.01 |

**Data Protein Mix assay LC MS**

Table S4: Results of the T4-Protein-Mix-Assay with LC-MS measurement. T4 and IS area counts are depicted alongside the recovery calculated based on the calibration line (data not shown) and the T4 concentration normalized to the negative controls C1.1-C1.5. Samples S2.3 and S3.1 could not be measured due to technical errors.

|  | Concentration Test item [nM] | Area T4 [counts*min] | Area 13C-T4 [counts*min] | Recovery [%] | Normalized T4 concentration [%] |
| --- | --- | --- | --- | --- | --- |
| C1.1 | 0 | 19943.401 | 16424.841 | 109.719 | 100.938 |
| C1.2 | 0 | 18116.476 | 17611.578 | 92.952 | 85.513 |
| C1.3 | 0 | 20424.971 | 17436.61 | 105.848 | 97.377 |
| C1.4 | 0 | 21835.041 | 16577.948 | 119.016 | 109.492 |
| C1.5 | 0 | 18918.96 | 14742.629 | 115.959 | 106.679 |
| C3.1 | 100000 | 16.475 | 17880.791 | 0.083 | 0.077 |
| S1.1 | 10 | 18668.692 | 16819.117 | 100.298 | 92.272 |
| S1.2 | 10 | 16447.948 | 15625.427 | 95.118 | 87.506 |
| S1.3 | 10 | 21069.748 | 17160.029 | 110.949 | 102.070 |
| S2.1 | 100 | 24979.454 | 15671.338 | 144.032 | 132.506 |
| S2.2 | 100 | 24502.918 | 15631.071 | 141.649 | 130.313 |
| S2.3 | 100 | n.a | n.a. | n.a | n.a. |
| S3.1 | 500 | n.a. | n.a. | n.a. | n.a. |
| S3.2 | 500 | 23265.599 | 16423.837 | 128.004 | 117.760 |
| S3.3 | 500 | 24606.864 | 16947.041 | 131.204 | 120.704 |
| S4.1 | 1000 | 23678.45 | 16428.358 | 130.239 | 119.817 |
| S4.2 | 1000 | 23122.727 | 16476.064 | 126.814 | 116.666 |
| S4.3 | 1000 | 21745.478 | 17426.194 | 112.759 | 103.735 |
| S5.1 | 2500 | 21788.566 | 16520.963 | 119.173 | 109.636 |
| S5.2 | 2500 | 21486.872 | 16303.718 | 119.088 | 109.558 |
| S5.3 | 2500 | 21762.041 | 16726.44 | 117.565 | 108.157 |
| S6.1 | 5000 | 19850.334 | 16479.908 | 108.842 | 100.132 |
| S6.2 | 5000 | 21081.306 | 16085.512 | 118.426 | 108.948 |
| S6.3 | 5000 | 20221.651 | 17121.13 | 106.725 | 98.184 |
| S7.1 | 10000 | 19031.716 | 15861.709 | 108.420 | 99.744 |
| S7.2 | 10000 | 16820.022 | 14721.274 | 103.244 | 94.982 |
| S7.3 | 10000 | 18466.88 | 15837.493 | 105.364 | 96.932 |
| S8.1 | 100000 | 13976.838 | 17120.31 | 73.770 | 67.866 |
| S8.2 | 100000 | 13703.657 | 17263.713 | 71.727 | 65.987 |
| S8.3 | 100000 | 14073.166 | 18003.179 | 70.636 | 64.983 |
